# Human JCV Infections as a Bio-Anthropological Marker of the Formation of Brazilian Amazonian Populations

**DOI:** 10.1371/journal.pone.0046523

**Published:** 2012-10-12

**Authors:** Izaura M. V. Cayres-Vallinoto, Antonio C. R. Vallinoto, Vânia N. Azevedo, Luis Fernando Almeida Machado, Marluísa de Oliveira Guimarães Ishak, Ricardo Ishak

**Affiliations:** Laboratório de Virologia, Instituto de Ciências Biológicas, Universidade Federal do Pará, Belém, Pará, Brasil; Centers for Disease Control and Prevention, United States of America

## Abstract

*JC polyomavirus* (JCV) is a member of the *Polyomaviridae* family. It presents a tropism to kidney cells, and the infection occurs in a variety of human population groups of different ethnic background. The present study investigated the prevalence of JCV infection among human populations from the Brazilian Amazon region, and describes the molecular and phylogenetic features of the virus. Urine samples from two urban groups of Belém (healthy subjects), one Brazilian Afro-descendant “quilombo” from the Rio Trombetas region, and native Indians from the Wai-Wai, Urubu-Kaapor, Tembé, Assurini, Arara do Laranjal, Aukre, Parakanã, Surui and Munduruku villages were investigated for the presence of the virus by amplifying VP1 (230 bp) and IG (610 bp) regions using a polymerase chain reaction. Nucleotide sequences (440 nucleotides, nt) from 48 samples were submitted to phylogenetic analysis. The results confirmed the occurrence of types A (subtype EU), B (subtypes Af-2, African and MY, Asiatic) and C (subtype Af-1) among healthy subjects; type B, subtypes Af-2 and MY, among the Afro-Brazilians; and type B, subtype MY, within the Surui Indians. An unexpected result was the detection of another polyomavirus, the BKV, among Afro-descendants. The present study shows, for the first time, the occurrence of JC and BK polyomaviruses infecting humans from the Brazilian Amazon region. The results show a large genetic variability of strains circulating in the region, infecting a large group of individuals. The presence of European, Asiatic and African subtypes associated to the ethnic origin of the population samples investigated herein, highlights the idea that JCV is a fairly good marker for studying the early migration of human populations, reflecting their early and late history. Furthermore, the identification of the specific mutations associated to the virus subtypes, suggests that these mutations have occurred after the entrance of the virus in the Amazon region of Brazil.

## Introduction

JCV is the etiologic agent of progressive multifocal leucoencephalopathy (PML), a progressive neurological disease of the central nervous system [1]–[4]. The emergence of HIV-1 elicited an additional interest in JCV with the frequent finding of HIV-1 carriers infected with JCV [4].

JCV infects the human host and establishes a persistent replicative cycle (including latency) following the acute infection, which favours its dissemination within human populations and its carriage through generations. It rarely appears to produce recognizable disease. Seroepidemiologic studies have shown that 70% to 80% of the adult human population shows the presence of antibodies to JCV, usually in the absence of clinical symptoms [1], [3], [5], [6]. It is a common thought that the primary infection probably occurs early in childhood [7], and the virus persistently replicates in renal cells with a continuous excretion through the urine [8], [9]. Thus, dissemination of JCV may be accomplished both in a horizontal and vertical manner.

The ancestral JCV probably generated three phylogenetic groups, designated as types A, B and C, which further mutated into subtypes that were distributed across the Old World, infecting human populations groups who were already there or were introduced into the geographic areas [10]. The divergence within genomes of DNA groups was estimated to reach 1–2.5% while the sequences of subtypes vary from 0.5–1% [11]–[14].

Population studies based on the detection of viral DNA in urine shows the presence of JCV ranging from 20% in Africa to 65% or more in Asia and in Asia-descendant population groups [15]. The major genetic diversity is currently described in Asia, where most of the subtypes are found, including B1-a, B1-b, d, B1, B2, B3, CY, MY, and SC [14], [16]. JCV is frequently used as a biomarker to trace the pattern of ancient and recent human migration. Its comprehensive geographic and human distribution provides a quite rigorous and safe method to associate human ethnic characteristics, social, commercial and cultural factors which influenced different human population groups through their history [15].

The present study aimed to describe the occurrence of JCV, including their types and molecular subtypes among human populations in the Brazilian Amazon and their distribution according to their ethnic background.

## Materials and Methods

### Populations Examined

**Figure 1:**
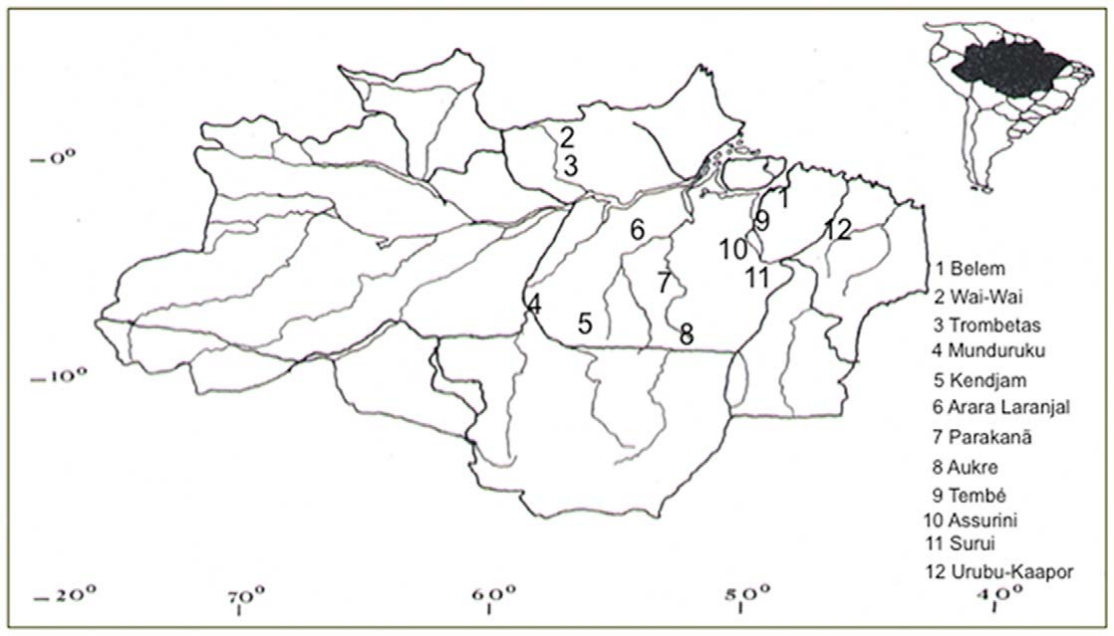
Geographical location of urban and non-urban populations investigated in the present study.

Urine samples from three groups were examined: urban (341 individuals residing in Belem, Para), Amerindians (42 individuals from 10 native Indian tribes from the Arara do Laranjal, Assurini do Trocara, Aukre, Kendjam, Munduruku, Parakanã, Surui, Tembe, Urubu-Kaapor and Wai-Wai villages) and 63 Afro-descendants living in the region of Rio Trombetas. Table 1 describes further demographics characteristics and Figure 1 points their geographical localization.

**Table 1:** Demographic information from the population groups examined for the presence of JCV infection.

Variables	Urban	Amerindians	Afro-descendants
Total examined	341	42*	63**
Males	154	3	38
Females	187	38	23
Age range (yo)	1–96	14–58	1–69

* One individual did not have sex registered;

** Two individuals did not have sex registered.

### Ethical considerations

The individuals were briefed about the project and those who accepted to take part were given an inform consent to sign. The present work was submitted and approved by the Ethics Committee of the University Hospital Joao de Barros Barreto and followed the Brazilian Guidelines and Regulatory Standards for Research Involving Human Subjects (Resolutions 196/1996 and 347/2005 of the Brazilian National Health Council).

### Urine Sample Collection and DNA extraction

Urine samples (50 mL) were collected in vial collectors and transported to the Virus Laboratory of the ICB/UFPA and stored at −20°C until assaying for JCV infection. The urine samples were centrifuged at 5,000 rpm for 15 minutes, the urinary sediment washed three times with sterile saline solution and the cell pellet used to DNA extraction following the protocol using the EZ-DNA kit, Gentra Systems, Inc., USA.

### Molecular analysis

Polymerase Chain Reaction - PCR was performed for amplification of the VP1 gene (215 bp) and the genomic region IG (610 bp) of the JCV, using a thermocycler (Mastercycler Personal, Eppendorf, Germany). The amplified products were subjected to purification for subsequent sequencing analysis of the nucleotide bases.

The reactions for the amplification of both segments (215 and 610 bp), were performed in a volume of 50 µL containing 400 ng of DNA extracted, 200 µM of each dNTP, 20 pmol of each primer, 50 mM KCl, MgCl_2_ 2.0 mM, Tris-HCl pH 8.3 10 mM and 0.5 U of Taq DNA polymerase. The pair of primers involved for segment 215 bp was: (JLP-15-F) 5′-ACAGTGTGGCCAGAATTCACTACC-3 and (JLP-16-R) 5′-TAAAGCCTCCCCCCCAACAGAAA-3′, corresponding to nucleotides (nt) 1710–1734 and 1924–1902 of the JCV genome [13]. Amplification reaction included an initial denaturation, at 95°C for 5 minutes, followed by 50 cycles of 95°C (1 min.), 63°C (1 min.) and 72°C (1 min.), and a final extension of 10 minutes at 72°C. The pair of primers involved for segment 610 bp was: (P-1-F) 5′-TTTTGGGACACTAACAGGAGG-3′ and (P-2-R) 5′-AGCAGAAGACTCTGGACATGG-3′, corresponding to nucleotides 2107–2127 and 2762–2742 of the JCV genome [17]. Amplification reaction included an initial denaturation, at 94°C for 5 minutes, followed by 50 cycles of 94°C (90 sec.), 55°C (90 sec.) and 72°C (150 sec.), and a final extension of 10 minutes at 72°C.

The products of amplification were visualized after electrophoresis in agarose gel with 2% in TAE 1× buffer containing ethidium bromide and the aid of a transilluminator with a source of ultra-violet light. Purification of the products followed the protocol of the QIAquick PCR Purification Kit (Qiagen, Inc., USA).

Sequencing and phylogenetic analysis - The amplified products of the IG region were used for the construction of the phylogenetic trees and subsequent relationships among the strains detected. Fourty-nine samples were sequenced according to the quality of the amplified product which was generated. The products with a reasonable quantity provided good sequences suitable for the phylogenetic analysis. The amplified fragments were submitted to a direct sequencing assay (both forward and reverse) according to the protocol of the ABI Prism Dye Terminator Cycle Sequencing Ready Kit (Applied Biosystems, US) and the products were loaded on the ABI Prism 310 DNA Sequencer (Applied Biosystems, US). The nucleotide sequences were used together with other JCV sequences available in the Genbank (HR5, C24704; HR13, C24711; AT-2, C24690; AT-4, C24692; SD-9, C24753; IT-8, AU078543; KO-2, AU078521; KO-3, AU078520; KO-5, AU078518; ES-3, AU078596; IT-5, C22883; G2, AU078596; IT-2, C22881; SP-7, AU078565; N5, C22891; UK-2, C22875; G4, AB074580; GK-3, AB048563; SP-1, C22878; UK-1, AB004499; SW-3, C22886; MR-7, C22873; N25, AU078585; G5, C22894; IT-3, C22882; AM-18, AU078447; SI-1, AU078574; SI-7, AU078568; GH-2, D43780; GH-4, D43782; GH-3, D43781; GH-1, D43779; KE-1, C22847; ET-3, C22844; ET-1, AB004469; IN-1, C22790; SU-5, AB127019; GR17, AU078479; UZ-24, AB061161; NG-2, AB127004; SO-1, C22841; CW-2, C22715; ML-6, AB048581; ID-1, C22796; MO-11, C22761; AT-8, C24696; MY, AU062350; Tokyo-1, AF030085; TKY-1, AB038254; HR-7, C24706; MU-3, C22865; IN-6, C22794; MU-9, C22868; MO-2, C22755; MO-5, C22758; MO-3, C22756; SL-2, C22814; CB-3, C22747; MO-6, C22759; MO-1, AB048561; CY, JQ237149; ML-1, C22805; CB-2, C22746; C2, C22721; JS-K, CO897282; GS-B, AF004350; N4, C22890; SA-5, C22818 and SA-3, C22817. The sequences were alligned using the Phylip 3.56 software package [18] in order to construct phylogenetic trees using the Neighbour Joining (NJ) method trees. The statistical reliance of the NJ tree was evaluated using 1,000 bootstrap samples. The trees were drawn with the TreeView 1.4 program [19]. Nucleotides sequences obtained in the present study are available in the GenBank databases under the accession numbers JQ947987 to JQ948035.

## Results

**Figure 2:**
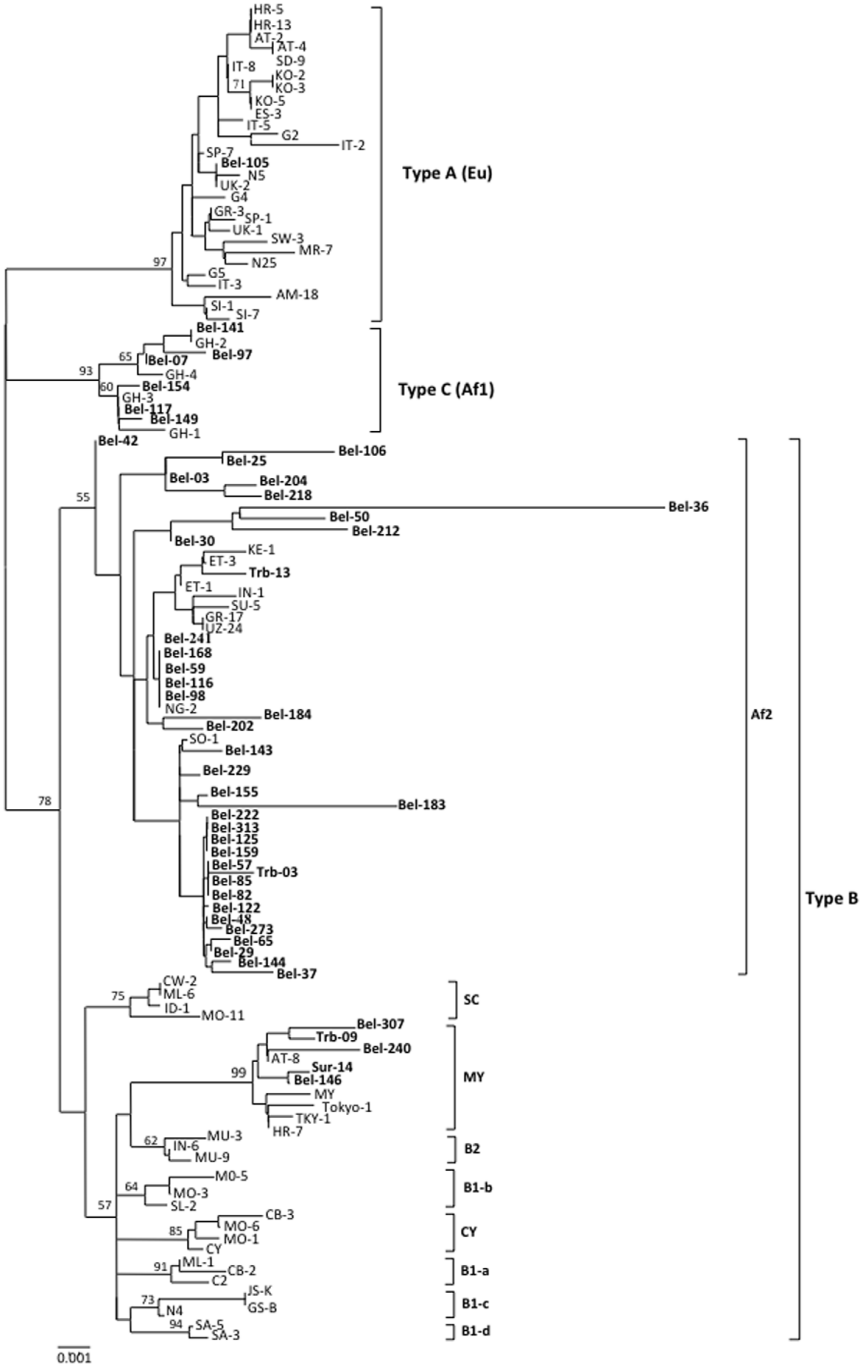
Unrooted phylogenetic tree showing the relationships of JCV strains isolated in the present study, including those described in the GenBank. Tree was constructed using the Neighbor-Joining method after alignment of 440 nucleotides of the genomic region IG. Statistical support was performed by using 1,000 bootstrap.

The geographical distribution of native Indian tribes and the African-descendants of original “quilombos” from the State of Pará (Trombetas) in relation to the city of Belém is depicted in Figure 1. The prevalence of JCV was described according to the cumulative amplification of VP1 and/or IG regions. One third (113/341) of the urban samples elicited both genes (87) or solely the VP1 region (26). Most of the infected individuals (66%) were older than 30 years. Phylogenetic analysis of 49 samples, showed the occurrence of Types A (3%), B (83%) and C (14%; Figure 2). The Amerindians consisted mostly of women (90.5%), younger than 40 years old (85.7%) and JCV (type B) infection was detected in one (2.4%) woman (older than 40 years) from the Surui tribe. The African descendants distribution was similar according to sex, mean age over 30 years old and 40% (25/63) were found to be infected. Four samples amplified both VP1 and IG regions, while 21 samples amplified VP1 solely. JCV type B was detected in three of the samples.

Additionally, the molecular analysis showed the amplification of a nucleotide sequence of 579 bp, characteristic of BKV poliomavírus, showing 98.9% of identity with the PittVR4 line in one sample of the Trombetas “quilombo”.

Phylogenetic trees (Neighbor-Joining) constructed using the genomic sequences of the IG region (Figure 2), showed the occurrence of three types of JCV (A, B and C) in samples of the urban population of Belém.

Type B was mostly of Af2 subtype. Four samples of type B were classified as subtype MY (Asia), with 99% of bootstrap. These samples showed 99% similarity with the prototype MY (ME-4/MYd) described by Zheng et al. [14] for North American natives.

The samples grouped as Type C (Type 6) belonged to subtype Af1 (African). This group presented the bootstrap value of 93%. A sample of the urban population of Belem was characterized as belonging to Type A subtype EU (bootstrap of 97%). The African-descendant samples of the Trombetas, elicited JCV strains Type B, subtypes MY and Af2. The analysis of a single sample of an indigenous community (tribe Surui), showed the presence of Type B subtype MY, also equivalent to the prototype MY (ME-4/MYd) described by Zheng et al. [14] for North American natives.

The analysis of the sequences (data not shown) enabled the identification of specific mutations among viral subtypes. The samples belonging to type B, subtype MY presented mutations in specific positions: 2242 nt (C→A/G), 2285 nt (C→G), 2309 nt (A→G), 2362 nt (A→G) and 2407 nt (C→T). The samples grouped as Type C showed a T→G mutation at position 2353 nt. Moreover, the samples grouped in Type B, subtypes MY and Af2 showed mutations which were common to these subtypes at positions 2312 nt (G→A) and 2377 nt (T→C).

## Discussion

The present investigation is probably the first one among apparently healthy individuals in Brazil, as well as, among native Indians and Afro-descendant groups (originated from escapee slaves during the XIX century) in the North region of the Brazilian Amazon region.

The sex composition was balanced and followed the demographics of the city of Belem, the largest and most important city of the North region of the country [20]. Several available studies do not inform the ages of the infected persons, except for some studying population groups from Africa [21], North America [11], [22], [23], Europe [2], [13], Australia [24], [25] and North Korea [26]. It is believed that primary infection occurs early in childhood [27], consequently, it is necessary to keep monitoring the infection among different age groups of different populations, healthy or not, once JCV is a persistent infection which is largely disseminated among human groups [1], [3], [5], [6].

Among the Afro-descendants and the residents in Belem, older than 30 years of age, 65% and 66%, respectively, were infected, similarly to what has been described when testing new diagnostics kits, in Australia and among persons with non-Hodgkin lymphoma [2], [25], [28]. Although there are few studies among young individuals, the number of samples is low and the available volume of urine does not favour the amplification of DNA [21], [26], [29].

JCV infection was defined by the detection of two genetic codifying areas (VP1 and IG regions), which present higher heterogeneity as compared to the regulatory region. The use of molecular biology assays is in agreement with seroepidemiological studies to determine the prevalence of the virus [15], [21], [25], [26], [30], [31]. Most of the results consider the presence of IG [7], [9], [10], [14], [15], [32], but its size (610 bp) is not advantageous and may compromise the results [26], [30], [33]. The use of a smaller region (VP1) is equally useful to describe the occurrence and the estimates of prevalence rates of infection by JCV among human populations [21], [24], [25].

In the present paper it was possible to amplify both regions in 77%, 52% and 16% of samples from Amerindians, urban groups and Afro-descendants, respectively. The efficiency of amplification of IG is highly dependent on the supercoiling nature of polyomaviruses [33]. Another possibility may be the small number of copies of DNA present in the samples, as well as, the small amounts of urine available for the isolation of DNA, which could lead to low levels of detection [26], [34]. The lower levels of prevalence found among the samples originated from Afro-descendants might have had some inadequacies during transportation regarding the time elapsed from collection to arrival in the laboratory and the conditions of temperature during transportation. Such conditions might prevent more significant interpretations of the frequency rate found in the group.

The prevalence of JCV among residents in Belem (33%) is comparable to those found in Australia (29%) [24], among urban populations in the USA (40%) [22], and among European countries (range from 30% to 44%) [13], [32]. It is common to find low prevalence rates among groups residing in South California, USA (18%) [7], North Korea (20%) [26] and Norway (21%) [30], as well as higher ones in the Philipines (47%) [16] and among Japanese descendants residing in the USA (53%) [7].

The presence of JCV in one Surui Indian woman is a good indicator that the virus may be circulating among the community as they still are an epidemiologically semi-closed population. The small number of individuals examined may have accounted for the sole finding. A large variation of prevalence rates has been reported in the Americas that includes the Inuits in Canada (26%) [10], among the Navahos (66%) and the Flathead (56%) in the USA, rates which were similar to those found (69%) in the Chamorro Pacific, Micronesia [11], in Asian populations (65%) [15], in Australia (33% in urban groups and 28% among natives) [24], [25] and within native populations from Siberia, with rates of excretion ranging from 13% (Chuckchis), 35% (Luskys and Yukaghirs), 39% (Koryaks) to 56% in the Nanais [10].

JCV infections among Afro-descendants is usually similar. The prevalence among pigmies was 22% and 20% among Bantus [21]. An average of 20% was found among other groups of the continent [15], a figure slightly lower than that described in the present paper (40%) and for Afro-Americans (56%) in the USA. The difference may be attributable to the intense miscigenation of Afro-descendants in Brazil and in the USA.

JCV is usually associated to population groups and their migration patterns in the same fashion as with mitochondrial DNA and the Y chromosome [35]. There is a strong correlation observed between the multiple viral subtypes and the different population groups, according to their ethnical ancestry [13], [14], [16].

In Belem, three ancestral types were described, according to its geographical association to the type A (3%), found in Europe, type B (83%), found in Africa and Asia, and type C (14%) found in the African continent. This is in strict adherence to the historical process of formation of the population of urban communities like Belem, as well as to the genetic information available of the composition of the urban population of Belem using classic genetic polymorphisms [36], and molecular genetic markers [37].

Type B was the most frequently found and samples from Belem which were subtyped as Af2 (66%) were mostly from “mesticos” (84%) who were born in the city as well (56%). It is relevant to mention that 25% of the individuals presented in their demographic records the observation of phenotype characteristics related to a black ethnicity, but without a self referral as such. This is a relevant matter when considering that most of the Af2 genotype evolved within the African continent approximately 50,000 years ago [35]. The results show both the African origin of JCV subtype and the origin of the individuals present in a high genetically mixed population.

All samples from Belem classified as subtype MY were originated also from individuals classified as “mesticos”. Two were born in Belem and two were born in two different villages. The samples showed 99% similarity to the MY prototype described among native Americans [14]. The samples originated from the “quilombo” populations, two were of subtype Af2 and one of subtype MY. The Surui Indian sample was equivalent to the MY subtype described within native Americans [14].

The high similarity observed among the samples from urban areas, the native Indians and the prototype isolated from native Americans is suggestive that type B, subtype MY, entered into the Brazilian Amazonian population during its process of formation by the mixture of migration patterns more than 10,000 years associated to the introduction of the European and the incoming African groups.

JCV is shown herein, for the first time, as a strong marker of population migration patterns when the similarity of the American strains are compared, including those of epidemiologically closed communities originated after the crossing of the Behring Strait and settling in the Americas [11], [15]. JCV, type B, subtype MY, establishes persistence in the human host and was brought by the migratory Amerindians and later disseminated to the present trihybrid populations residing in the area. Once it was present among the native Indians in the Amazon region, subtype MY could have entered into the original “quilombos” (slave communities formed after their fled from slavery in cities, villages and farms). “Quilombos” date as back as 1788 in the State of Para, Brazil [38] and their number increased markedly from the end of the XVIII to the beginning of the XIX centuries. Some genetic studies strongly support this view of ethnical mixture [39], [40].

In a similar way, the strain was introduced in the city of Belem probably from its origin in 1616 due to the extensive mixture of the three ethnical groups. The contribution of the Indian population within urban areas is approximately 35% and to a lower extent, that of the Afro-descendants [36], [37]. Again, the most probable origin of the MY subtype is the link to the North American Indians.

The phylogenetic relationship among JCV strains from Japan, Korea and Amerindians suggests that emergence of subtype MY occurred between 50 and 30 thousand years ago and that within the MY clade between 30 and 10 thousand years [14]. The separation of genotypes associated to native American Indians has probably occurred some 15,000 years ago, an estimate which is in agreement with the archeological evidence for the entry of the first humans into the American continent. The proposed model is similar to that which occurred with another human persistently infecting virus, HTLV-2 [41].

The role of JCV as a marker of past and recent human migration and mixture of different ethnic groups is also defined by the detection of type C, in which all samples were identified as subtype Af1. The isolates were originated from individuals with phenotypic and socio demographic informations of being all “mesticos” of whom 60% resembled whites and 40% negros. Most of them were born outside large cities.

Type C represents the ancestral JCV that evolved circa 100 thousand years ago, generating subtype Af1, which was subsequently disseminated into West Africa, an area associated to the emergence of the human species, and later to Central Africa including Central African Republic, Ghana and Mauritania [35], [42]. The identification of type C, subtype Af1 among urban residents, again indicates the contribution of the African slaves to the genetic composition of the population. In a similar fashion as type B, subtype Af2, both are homogeneously distributed among the tri-hybrid groups of humans in the Amazon region of Brazil.

It has been proposed that the ancestral JCV emerged together with man (circa 200–100 thousand years ago) in Africa [35]. Type C, subtype Af1 is the probable prototype which first emerged, and later, circa 50,000 years, types A and B emerged. When the first humans started their migration patterns, JCV was also disseminated. One possible route, probably the oldest one, suggests that humans hosted JCV ancestral type A in their pathway towards outside Africa. A second migration route, probably took place within the African continent, and included humans hosting ancestral type B of JCV.

The first modification that took place in type B, originated Af2 and the non-Af2 subtypes. Subtype Af2 was then disseminated within the African continent and, secondarily, to Saudi Arabia, the West and South of Asia, as well as to Southeast Europe (circa 50 to 10 thousand years ago). The non-Af2 strains originated the B1-c, a minor European genotype, and the main genotypes present in Asia, Oceania and the Amerindians from North America (B1-a, B1-b, B1-d, B2, CY, MY, SC, 8A, 8B e 2E).

Type A, subtype EU was also detected within the urban population of Belem, from one individual whose relatives dated back to the routes of heavy migration from Portugal during colonization. This recent migration pattern of Europeans, particularly in the last 300 years, enables the role of JCV as a good marker to trace patterns of ancient and recent migration of humans and its genetic mixture with other ethnic groups.

Mutations in positions 2242 nt (C→A/G), 2285 nt (C→G), 2309 nt (A→G), 2362 nt (A→G) and 2407 nt (C→T), may be considered as a signature of the viral subtype after entering the Amazon region of Brazil. Maintenance of JCV is a consequence of persistence in the human host, its moderate genetical stability and the low levels of DNA mutations. The presence of the same pattern of mutations even within a woman from the Surui native Indians, a semi-closed epidemiological community, reinforces the genetic peculiarity of subtype MY in the Amazon region of Brazil and the transmission of the virus from the ancestral human native populations to the Afro-descendants and the present tri-hybrid human populations.

It was unexpected the finding of a nucleotidic sequence of the polyoma virus BK in an Afro-descendant individual, but it was previously described among patients who received bone marrow transplants [43], renal transplant recipients [44] and among Chinese healthy adults [29]. The amplification of VP1, is useful for the detection of JCV and BKV as well, as they share approximately 72% of nucleotide homology in the studied [6], [25], [45]. The presence of both viruses indicate the dissemination of polyomaviruses and the need to improve their clinical detection among human populations.
